# Transcriptome Analysis Unveils That Exosomes Derived from M1-Polarized Microglia Induce Ferroptosis of Neuronal Cells

**DOI:** 10.3390/cells11243956

**Published:** 2022-12-07

**Authors:** Sheng Gao, Shu Jia, Luyue Bai, Dongru Li, Chunyang Meng

**Affiliations:** 1 Department of Medicine, Qingdao University, Qingdao 266071, China; 2 Department of Medicine, Jining Medical University, Jining 272067, China

**Keywords:** microglia, exosomes, neuronal cells, transcriptome, ferroptosis

## Abstract

Microglia play a vital role in neurodegenerative diseases. However, the effects of microglia-derived exosomes on neuronal cells are poorly understood. This study aimed to explore the role of M1-polarized microglia exosomes in neuronal cells by transcriptome analysis. Exosomes isolated from resting M0-phenotype BV2 (M0-BV2) microglia and M1-polarized BV2 (M1-BV2) microglia were analyzed using high-throughput sequencing of the transcriptome. Differentially expressed genes (DEGs) between the two types of exosomes were identified by analyzing the sequencing data. The biological functions and pathways regulated by the identified DEGs were then identified using bioinformatics analyses. Finally, we evaluated the effects of exosomes on neuronal cells by coculturing M0-BV2 and M1-BV2 exosomes with primary neuronal cells. Enrichment analyses revealed that DEGs were significantly enriched in the ferroptosis pathway (*p* = 0.0137). M0-BV2 exosomes had no distinct effects on ferroptosis in neuronal cells, whereas M1-BV2 exosomes significantly reduced ferroptosis suppressor proteins (GPX4, SLC7A11, and FTH1) and elevated the levels of intracellular and mitochondrial ferrous iron and lipid peroxidation in neuronal cells. Polarized M1-BV2 microglia exosomes can induce ferroptosis in neuronal cells, thereby aggravating neuronal damage. Taken together, these findings enhance knowledge of the pathogenesis of neurological disorders and suggest potential therapeutic targets against neurodegenerative diseases.

## 1. Introduction

Microglia are resident macrophages in the central nervous system (CNS) and account for approximately 12–15% of the total CNS cellular population [1,2,3]. As the central nervous system’s first-line defense, microglia play a vital role in the occurrence and development of nerve injury and neurological disorders [4]. There is consensus that microglia are significantly associated with Alzheimer’s disease [5], Parkinson’s disease [6], and amyotrophic lateral sclerosis [7]. Previous studies have demonstrated that continuously polarized microglia play a major role in neurological disorders, especially through the effects of substances secreted by microglia [8,9]. Extracellular vesicles (EVs) derived from microglia can mediate the excitability of neurons and transfer biomolecules, such as proteins and RNAs, between different cells [10,11]. When internalized by the target cells, EVs can modify their phenotype. Exosomes refer to a type of EV measuring 30–100 nm that is actively secreted by cells [12]. Exosomes contain active substances, such as lipids, nucleic acids, and proteins, and release their contents after binding to target cell receptors, thereby regulating intercellular signal transduction pathways [13]. Given the established role of microglia in disease, it is important to explore their effects of microglia exosomes on neuronal cells and the underlying mechanisms. Recent studies have revealed that ferroptosis, a form of iron-dependent cell death involving severe lipid peroxidation [14], is associated with various neurological disorders [15]. Microglia can help maintain iron homeostasis and normal physiological functions in the brain [16]. In the present study, transcriptome analysis of exosomes secreted by microglia revealed an important pathological process: ferroptosis. Thus, we hypothesize that M1-polarized microglia induce ferroptosis of neuronal cells through secreted exosomes. To test this hypothesis, we evaluated the effects of exosomes on ferroptosis proteins, lipid peroxidation, and the ferrous iron levels of neuronal cells. At present, polarized microglia are known to release exosomes containing proteins related to neurodegenerative diseases, such as α-synuclein [17], cytokines [18], Aβ [19], and tau [20]; however, the effects of microglia exosomes on ferroptosis of neuronal cells remain unclear. Our findings further the knowledge on the mechanism of neuron–glia crosstalk in neurological disorders and demonstrate that M1-polarized microglia exosomes disrupt the intracellular homeostasis of iron and lipid oxidation to induce the ferroptosis of neuronal cells. These findings offer new insights into the pathogenesis of neurological disorders and suggest potential therapeutic targets for neurodegenerative diseases.

## 2. Materials and Methods

### 2.1. Culture and Polarization of BV2 Microglia

BV2 microglia were procured from Procell Life Science & Technology Co., Ltd. (Wuhan, China) and cultured in Dulbecco’s Modified Eagle Medium (DMEM) supplemented with 10% fetal bovine serum (FBS) and 100 U/mL penicillin/streptomycin at 37 °C under 5% CO_2_. The medium was refreshed every 2 days. When 80% confluence was attained, the resting M0-phenotype BV2 (M0-BV2) microglia were subcultured for further passage. M1-polarized BV2 (M1-BV2) microglia were obtained by induction of resting M0-BV2 with 1 µg/mL lipopolysaccharide (LPS; *Escherichia coli* LPS 055: B5, Sigma Aldrich, Saint-Louis, MO, USA) for 12 h. BV2 microglia are the most frequently used cell line for primary microglia, and their overall response pattern parallels that of primary microglia when triggered by LPS [21].

### 2.2. Identification of Polarized M1-BV2 Microglia

The polarization of the M1-phenotype in BV2 microglia was verified by the detection of the M1-phenotype biomarkers CD40 and INOS using flow cytometry analysis. The cells were incubated with CD40 (BioLegend, San Diego, CA, USA) and INOS (BioLegend, San Diego, CA, USA) at room temperature in darkness for 20 min and assessed using a flow cytometer (CytoFlex; Beckman Coulter, Inc., Brea, CA, USA) according to the manufacturer’s protocol. A panel of proinflammatory markers (IL-1β, IL-6 and TNF-α) was also detected to confirm the polarization of M1-BV2 microglia using quantitative reverse-transcription polymerase chain reaction (qRT-PCR). Briefly, total RNA was extracted from BV2 microglia using TRIzol Reagent (Invitrogen, Carlsbad, CA, USA) and reverse-transcribed to cDNA using RT Master Mix (Invitrogen, Carlsbad, CA, USA). QRT-PCR was performed using Ultra SYBR Mixture in a CFX-96 Touch PCR Detection system (Bio-Rad Laboratories, Inc., Hercules, CA, USA). The expression levels of IL-1β, IL-6, and TNF-α were normalized to that of the reference GAPDH and calculated using the 2^−ΔΔ Ct^ method. The primers used for qRT-PCR are reported in Table 1.

### 2.3. Extraction of Exosomes

First, the culture supernatant was collected and centrifuged at 300× *g* for 10 min at 4 °C. The supernatant obtained was further centrifuged at 2000× *g* for 10 min, and the resulting supernatant was collected and further centrifuged at 10,000× *g* for 30 min. After each centrifugation step, the supernatant was carefully collected to avoid contamination with cell debris and other sediments.

Subsequently, the final supernatant was collected and filtered using a 0.22 mm sterile filter (Millipore Sigma, Darmstadt, Hessen, Germany). This was transferred to a high-speed centrifuge tube and ultracentrifugation was performed at 100,000× *g* for 70 min at 4 °C to obtain the exosomes. Finally, the supernatant was discarded and the precipitated exosomes were resuspended in phosphate-buffered saline (PBS) solution.

### 2.4. Identification of Exosomes

The extracted exosomes were identified using the following three methods: transmission electron microscopy (TEM) was used for morphological analysis; nanoparticle tracking analysis (NTA) was used to determine particle size; and Western blotting was used to characterize exosome-specific markers (CD9, CD81, and Alix).

#### 2.4.1. Transmission Electron Microscopy Analysis

Exosome solution (10 µL) was placed on a Formvar carbon-coated grid and allowed to rest for 10 min. Excess exosomes were absorbed by filter paper. The sample was then stained with 1% uranyl acetate solution for 1 min, washed twice with distilled water, and dried using filter paper. The samples were then imaged by TEM (HT-7700, Hitachi, Germany).

#### 2.4.2. Nanoparticle Tracking Analysis

The number and size of particles in the exosome solution (dilution factor: 10,000) were determined at 405 nm with a laser using a ZetaView (PMX120-Z, Particle Metrix, Meerbusch, Germany) according to the manufacturer’s protocol. Photos were taken at a rate of 30 pieces/sec for 1 min. The movement of particles was then analyzed using ZetaView software (Version 8.05; Particle Metrix, Inning, Germany).

#### 2.4.3. Western Blotting for Exosomes

Exosomes (20 µL) were mixed with 10 µL of radio-immunoprecipitation assay (RIPA) lysis buffer (Beyotime, Shanghai, China) for 30 min on ice and then centrifuged at 12,000× *g* for 10 min. The supernatant (exosome protein solution) was collected. The protein concentration was determined using a BCA protein assay kit (Beyotime, Shanghai, China) according to the manufacturer’s instructions. Proteins (20 μg/lane) from BV2 cells, M0-BV2 exosomes, and M1-BV2 exosomes were loaded and separated using 12% sodium dodecyl-sulfate polyacrylamide gel electrophoresis (SDS-PAGE) gels (Bio-Rad Laboratories, Hercules, CA, USA). The proteins were then transferred to polyvinylidene fluoride (PVDF) membranes (Millipore, Boston, MA, USA) and incubated overnight with primary antibodies against exosome-specific marker proteins, namely CD9 (1:1000; ABclonal), CD81 (1:1000; ABclonal), and Alix (1:1000; CST), according to previous studies [22,23]. Subsequently, the membranes were incubated in HRP-conjugated secondary antibody (1:4000; goat anti-rabbit IgG, ABclonal) for 1 h. Lastly, the protein bands were exposed to ECL reagent (Beyotime, Shanghai, China) and visualized using a chemiluminescent detector (Tanon Science and Technology Co., Ltd., Shanghai, China).

### 2.5. RNA Isolation and High-Throughput Sequencing of Transcriptome

Total RNA was extracted from M0-BV2 and M1-BV2 exosomes using Invitrogen TRIzol LS reagent (Invitrogen, Carlsbad, CA, USA), followed by mRNA enrichment using oligo (dT) magnetic beads. The RNA was fragmented, first-strand cDNA synthesis was carried out by reverse transcription with random primers, and second-strand cDNA synthesis was performed with the addition of dUTP. The final library was generated after the repair of double-stranded cDNA ends, the addition of A, and PCR amplification. The quality of the constructed library was assessed using Agilent 2100 followed by sequencing with an Illumina Hiseq4000 sequencer.

### 2.6. Metabolic Activity, Differentially Expressed Genes, and Functional Enrichment Analyses

Differential analysis of metabolic activity between M0-BV2 and M1-BV2 exosomes was performed with a metabolism-specific tool, Metabolizer [24]. The edgeR [25] package (version: 3.32.0) in R software (version 4.0.0) was used to normalize the sequencing data and identify differentially expressed genes (DEGs). DEGs were identified using the thresholds *p* < 0.05 and |log2FC| > 0.585. Gene ontology (GO) analysis was used to annotate the cellular component (CC), molecular function (MF), and biological process (BP) function in which the DEGs were enriched. The Kyoto Encyclopedia of Genes and Genomes (KEGG) and gene set enrichment analysis (GSEA) were used to identify the pathways in which the genes were enriched. To predict significantly enriched functions and pathways regulated by DEGs in M0-BV2 and M1-BV2 exosomes, the GO and KEGG enrichment results were identified using a threshold of *p* < 0.05. The ferroptosis pathway showed significant enrichment (*p* = 0.0137).

### 2.7. Isolation and Culture of Primary Neuronal Cells

C57BL/6J neonatal mice were euthanized by decapitation, rinsed with 75% ethanol to minimize contamination, and then rapidly submerged in ice-cold 1 × PBS. The skulls were opened and the brains were removed for separation of the cerebral cortex. The tissue was cut into 1 mm^3^ pieces, washed with 1 × PBS, and then digested in a shaking machine with digestion buffer at 5% CO_2_ at 37 °C for 10 min. FBS was added to stop the digestion reaction. A straw was used to gently blow on the suspension until there was no lumped tissue. The tissue suspension was then filtered through 100-, 200-, and 400-mesh strainers. Next, the filtrate was centrifuged at 300× *g* for 5 min, and the supernatant was discarded. The sediment was resuspended with PBS and centrifuged at 100× *g* for 5 min. The cell sedimentation was resuspended with complete medium of neuron cells (Procell, Wuhan, China), plated on a poly-L-lysine precoated culture dish, and then cultured in an incubator at 37 °C under 5% CO_2_ for 24 h. Subsequently, the cells were cultured in complete medium of neuron cells containing cytarabine (5 µmol/L) for 48 h to inhibit the growth of non-neuronal cells. After 48 h, the medium was refreshed.

### 2.8. Identification of Primary Neuronal Cells

Primary neuronal cells were verified by the detection of neuron-specific nuclear (NeuN) and neuron-specific enolase (NSE) proteins, which are specific markers of neurons [26,27]. The expression of NeuN and NSE was detected using immunofluorescence. Briefly, primary neuronal cells were fixed with 4% paraformaldehyde for 15 min. After washing three times with PBS, cells were blocked with 10% FBS containing 0.5% Triton (Solarbio, Beijing, China) for 20 min. Then, the cells were incubated with NSE (1:100; CST) and NeuN (1:100; Abcam) primary antibodies at 4 °C overnight, followed by incubation with conjugated anti-rabbit secondary antibodies (Proteintech, Chicago, IL, USA) at room temperature for 1 h. Finally, cells were mounted on slides after staining with DAPI (Beyotime, Shanghai, China). The stained cells were imaged using a fluorescence microscope (Olympus BX53; Olympus Corporation; Tokyo, Japan).

### 2.9. Labeling of BV2 Microglia-Derived Exosomes

M0-BV2 and M1-BV2 exosomes were stained with 1 µM DiI (Invitrogen, Carlsbad, CA, USA) for 30 min and then washed with PBS at 100,000× *g* for 2 h to remove excess dye, according to previous studies [28,29]. Finally, the labeled exosomes were resuspended in PBS.

### 2.10. Coculture of Neuronal Cells with Exosomes

Neuronal cells were cocultured in six-well plates with M0-BV2 exosomes (M0-Exosome group) and M1-BV2 exosomes (M1-Exosome group). PBS-treated neuronal cells served as the control group. For the M0-Exosome group, when 60% confluence was attained, the medium was replaced to eliminate interference by any exosomes secreted by the neuronal cells, and M0-BV2 exosomes (10^9^ particles) were added. For the M1-Exosome group, the same amounts of M1-BV2 exosomes were added. The control group was treated with the same volume of PBS. Neuronal cells in the three groups were then cultured for an additional 24 h.

### 2.11. Internalization of Exosomes

Neuronal cells in the control, M0-Exosome, and M1-Exosome groups were stained with 1 µM Hoechst 33342 (Thermo Fisher Scientific, Waltham, MA, USA) for 30 min at 37 °C in a 5% CO_2_ incubator, as per the manufacturer’s instructions. Entry of DiI-labeled exosomes into the neuronal cells was visualized by laser confocal microscopy (Zeiss LSM800; Zeiss AG; Oberkochen, Germany).

### 2.12. Protein Extraction and Western Blot Analysis

Protein extraction of neuronal cells in the control, M0-Exosome, and M1-Exosome groups was carried out using RIPA (Beyotime, Shanghai, China). Protein concentration was determined using a BCA assay kit (Beyotime, Shanghai, China) according to the manufacturer’s instructions. For each group, protein (20 μg/lane) was loaded and separated by 12% SDS-PAGE (Bio-Rad Laboratories, Hercules, CA, USA). The separated proteins were then transferred to PVDF membranes with a current of 300 mA for 80 min, blocked using 3% bovine serum albumin (Beyotime, Shanghai, China) for 2 h, and incubated overnight with primary antibodies against GPX4 (1:1000; Abcam), SLC7A11 (1:1000; ABclonal), FTH1 (1:1000; ABclonal), and β-actin (1:5000; ABclonal). Subsequently, the membranes were washed three times in TBST and incubated in HRP-conjugated secondary antibody (1:4000; goat anti-rabbit IgG, ABclonal) for 1 h. The membranes were once again washed in TBST. The protein bands were then exposed to the ECL reagent (Beyotime, Shanghai, China) and visualized using a chemiluminescent detector (Tanon Science and Technology Co., Ltd.). The relative protein expression was quantified using ImageJ software (National Institutes of Health) with β-actin as the control.

### 2.13. Measurement of Ferrous Iron and Lipid Peroxidation

Given the relationship among ferroptosis, lipid peroxidation, ferrous iron, and microglia [14,30,31], we assessed the levels of lipid peroxidation and intracellular and mitochondrial ferrous iron in the control, M0-Exosome, and M1-Exosome groups.

#### 2.13.1. Measurement of Lipid Peroxidation

The lipophilic probe C11BODIPY581/591 (Thermo Fisher Scientific, Waltham, MA, USA) was used to detect lipid peroxidation. Fluorescence changes (i.e., shifts from red to green upon oxidation) indirectly reflect the oxidation of unsaturated fatty acids [32]. Neuronal cells in the control, M0-Exosome, and M1-Exosome groups were stained with 1 µM C11BODIPY581/591 and Hoechst 33342 (Thermo Fisher Scientific, Waltham, MA, USA) for 30 min at 37 °C in a 5% CO_2_ incubator and then assessed by laser confocal microscopy (Zeiss LSM800; Zeiss AG; Oberkochen, Germany) according to the manufacturer’s instructions. Lastly, data were quantified using ImageJ software (National Institutes of Health).

#### 2.13.2. Measurement of Intracellular and Mitochondrial Ferrous Iron

The intracellular ferrous iron level was determined using FerroOrange (Dojindo Molecular Technology, Kumamoto, Kyushu, Japan) according to the manufacturer’s protocol. Briefly, cells were digested with 0.25% trypsin-EDTA (Gibco, Grand Island, NY, USA) and centrifuged at 500× *g* for 3 min. The cell precipitate was then resuspended with HBSS, stained with 1 μM FerroOrange for 30 min at 37 °C in darkness, and assessed using a flow cytometer (CytoFlex; Beckman Coulter, Inc.). To measure the mitochondrial ferrous iron level, the mitochondria, mitochondrial ferrous iron, and nuclei of neuronal cells were stained with 1 μM Mito-Tracker Red (Dojindo Molecular Technology, Kumamoto, Kyushu, Japan), 1 μM Mito-FerroGreen (Dojindo Molecular Technology, Kumamoto, Kyushu, Japan), and Hoechst 33342 (Thermo Fisher Scientific, Waltham, MA, USA) for 30 min at 37 °C in a 5% CO_2_ incubator. The level of ferrous iron in mitochondria was then assessed by laser confocal microscopy (Zeiss LSM800; Zeiss AG; Oberkochen, Germany) and quantified using the ImageJ software (National Institutes of Health).

### 2.14. Statistical Analysis

All the data are reported as the mean ± standard deviation (SD) from three independent experiments. Data were compared between groups using one-way analysis of variance (ANOVA). Differences of *p* < 0.05 were deemed statistically significant. Statistical analysis was performed using GraphPad Prism (Version 8.0.2, GraphPad Software, Inc., La Jolla, San Diego, CA, USA).

## 3. Results

### 3.1. Characterization of Polarized M1-BV2 Microglia and Exosomes

**Figure 1:**
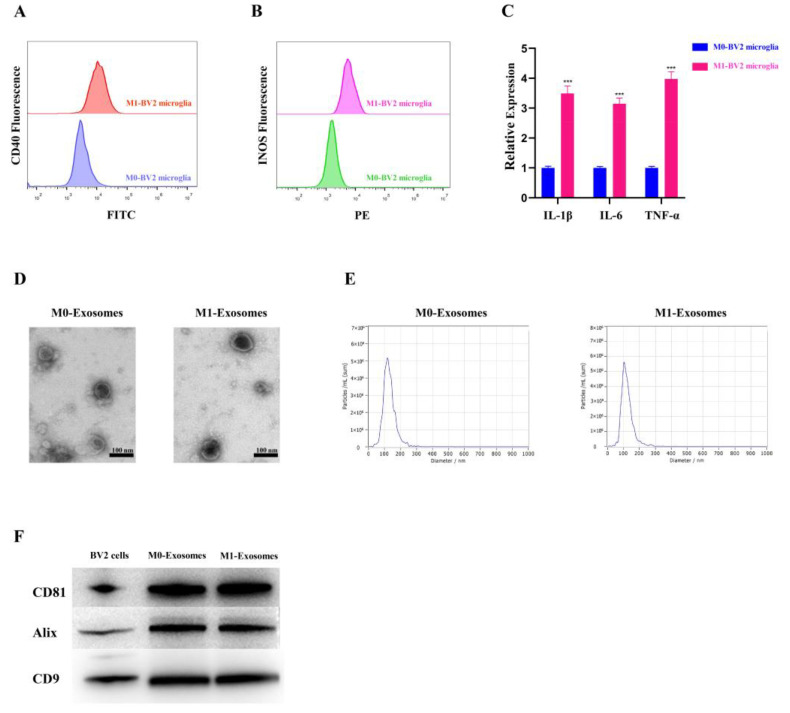
The characterization of polarized M1-BV2 microglia and exosomes. (**A**,**B**) Flow cytometry results showing expression of CD40 and INOS markers in BV2 microglia. (**C**) Relative expression of IL-1β, IL-6, and TNF-α in BV2 microglia (*** *p <* 0.001). (**D**) Transmission electron microscopic images of extracted M0-BV2 and M1-BV2 exosomes. (**E**) Diameter of the extracted M0-BV2 and M1-BV2 exosomes. (**F**) Measurement of the exosome-specific proteins CD81, Alix, and CD9 by Western blot. Data are presented as the mean ± SD of three independent experiments.

The flow cytometry results showed significant expression of the M1-phenotype markers CD40 and INOS in polarized M1-BV2 microglia (Figure 1A,B). QRT-PCR also demonstrated higher expression of IL-1β, IL-6, and TNF-α in polarized M1-BV2 compared to M0-BV2 (Figure 1C). Together, these results indicated successful polarization of M1-BV2 microglia. TEM revealed characteristic double-layer membranes in the extracted M0-BV2 and M1-BV2 exosomes (Figure 1D). According to the NTA results, the diameter of extracted BV2 microglia exosomes was approximately 100 nm, which is consistent with the expected diameter range (Figure 1E). Western blot analysis indicated enrichment of exosome-specific proteins in the extracted BV2 microglia exosomes (Figure 1F). Taken together, these results indicate successful exosome extraction.

### 3.2. Transcriptome Metabolic Analysis and DEG Identification

**Figure 2:**
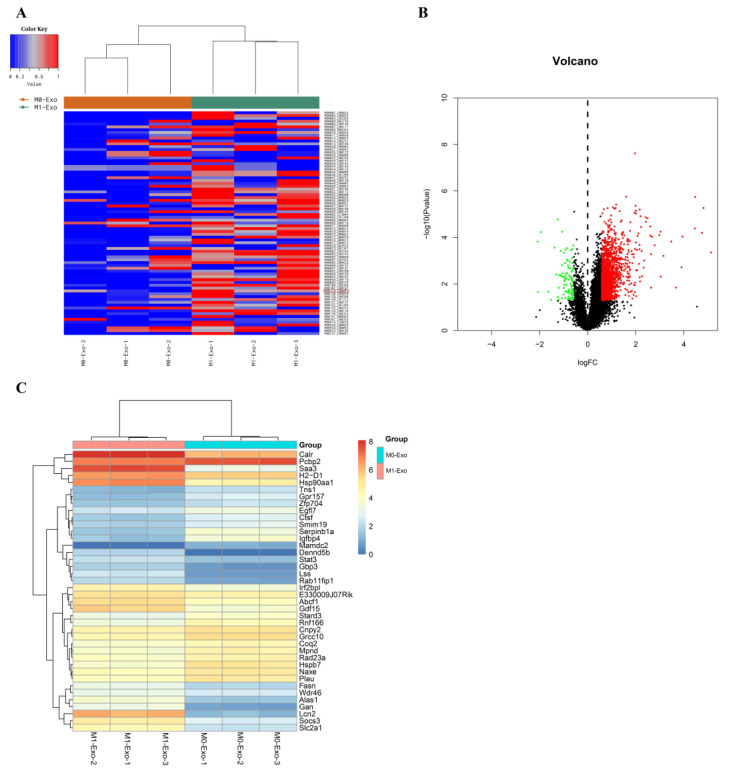
Differential analysis of metabolic activity and genes in M0-BV2 and M1-BV2 exosomes. (**A**) The heatmap of metabolic activity analysis between M0-BV2 and M1-BV2 exosomes. M00118_C00051 (red mark) is glutathione metabolism. (**B**) Volcano plot of upregulated (*n* = 1557, red) and downregulated DEGs (*n* = 102, green) in M0-BV2 and M1-BV2 exosomes. The black dots mean non-difference. (**C**) The heatmap shows the expression patterns of DEGs (top 40) in M0-BV2 and M1-BV2 exosomes.

Differential analysis of metabolic activity revealed distinct differences between M0-BV2 and M1-BV2 exosomes in glutathione metabolism (M00118_C00051; Figure 2A; red mark), which is an important mechanism of ferroptosis [33,34]. Transcriptome sequencing analysis of M0-BV2 and M1-BV2 exosomes resulted in 10036 genes (Table S1). Differential analysis identified 1557 upregulated genes (Table S2) and 102 downregulated genes (Table S3 and Figure 2B). The expression patterns of the DEGs are displayed as a heatmap (Figure 2C).

### 3.3. GO, KEGG, and GSEA Analyses

**Figure 3:**
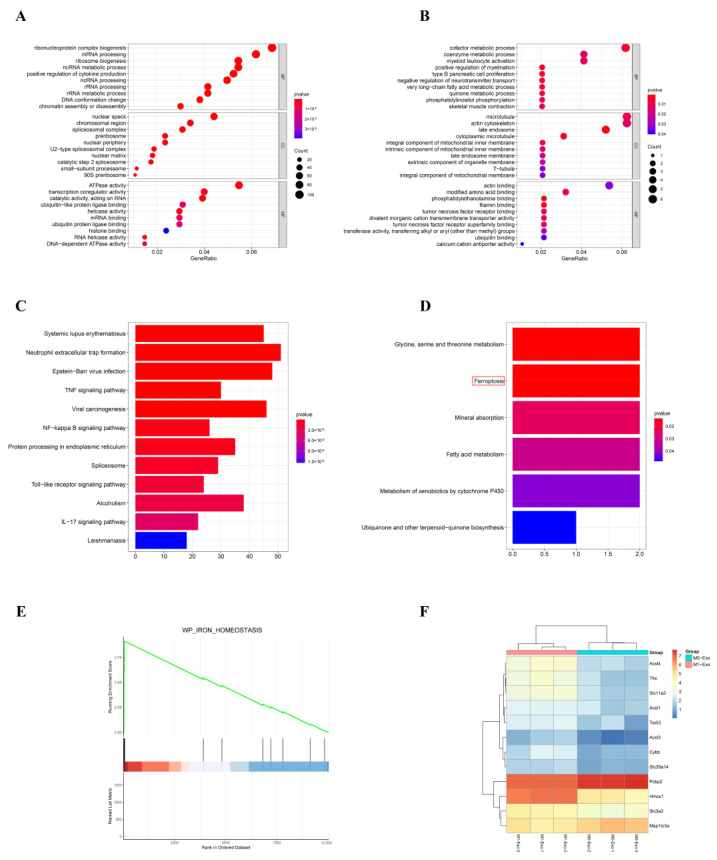
Results of the GO, KEGG, and GSEA enrichment analyses. (**A**,**B**) The top 10 enriched GO items (BP, CC, and MF) of upregulated and downregulated DEGs. (**C**) The top 10 pathways identified in which upregulated DEGs were enriched. (**D**) The ferroptosis pathway was prominently enriched (red mark). (**E**) Iron homeostasis was enriched in GSEA results. (**F**) A comparison of DEG expressions in ferroptosis pathway.

The results of GO and KEGG analyses of upregulated and downregulated DEGs are shown in Figure 3A–D. The DEGs were prominently enriched in the ferroptosis pathway (*p* = 0.0137; Figure 3D; red mark). In addition, iron homeostasis (Figure 3E), which is closely related to ferroptosis, is enriched in the GSEA results. A comparison of DEG expressions in the ferroptosis pathway is shown in Figure 3F.

### 3.4. Characterization of Neuronal Cells and the Internalization of Exosomes by Neuronal Cells

**Figure 4:**
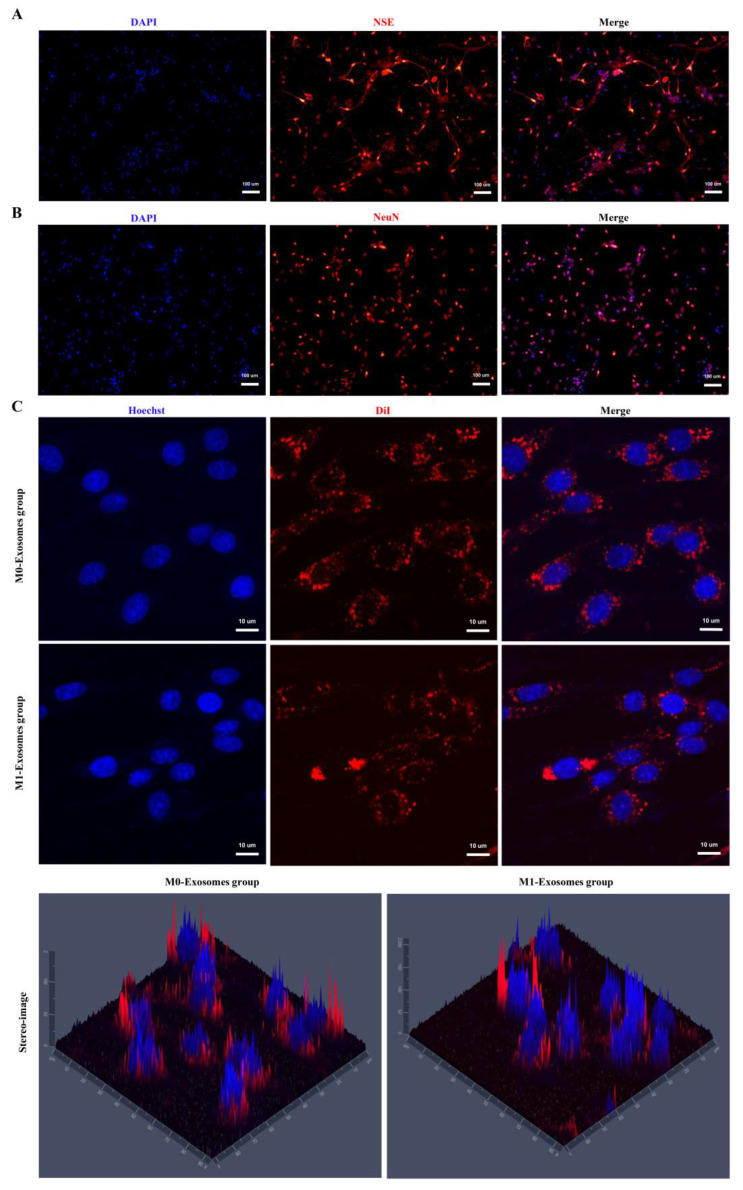
Molecular characterization of primary neuronal cells and the internalization of exosomes. (**A**,**B**) Immunofluorescence results showing the expression of NSE and NeuN in primary neuronal cells. Scale bars = 100 μm. Neuronal cells were stained with NSE (red) or NeuN (red), and the nuclei were stained with DAPI (blue). (**C**) Representative confocal images of DiI-labeled exosomes taken up by neuronal cells. Scale bars = 10 μm. M0-BV2 and M1-BV2 exosomes were stained with DiI (red), and the nuclei of neuronal cells were stained with Hoechst (blue).

Neuronal cells were characterized by the detection of neuron-specific markers (NSE and NeuN). The immunofluorescence results of NSE and NeuN in primary neuronal cells are shown in Figure 4A,B. These results indicate successful isolation of primary neuronal cells. The confocal images of DiI-labeled exosomes confirmed that BV2 microglia exosomes were taken up by neuronal cells (Figure 4C).

### 3.5. M1-BV2 Exosomes Regulate the Expression of Ferroptosis Proteins

**Figure 5:**
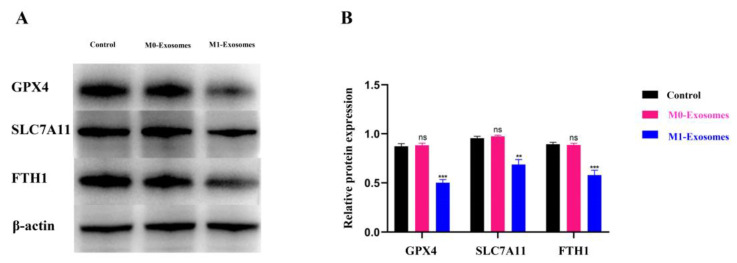
Levels of ferroptosis proteins in neuronal cells following different treatments. (**A**) Expression of GPX4, SLC7A11, and FTH1 in the control, M0-Exosome, and M1-Exosome groups was measured by Western blot. (**B**) Quantitative protein results from Western blots (ns *p* > 0.05, ** *p* < 0.01, *** *p* < 0.001). Data are presented as the mean ± SD of three independent experiments.

Given the significant enrichment of the ferroptosis pathway, ferroptosis marker proteins in neuronal cells were measured by Western blot following different treatments. Neuronal cells treated with M0-BV2 exosomes showed no significant alterations in ferroptosis protein levels relative to controls. In contrast, incubation with M1-BV2 exosomes significantly reduced levels of ferroptosis-suppressor proteins GPX4, SLC7A11, and FTH1 in neuronal cells compared to the control group (*p* < 0.05; Figure 5A,B).

### 3.6. M1-BV2 Exosomes Increase Lipid Peroxidation and Ferrous Iron Levels in Neuronal Cells

**Figure 6:**
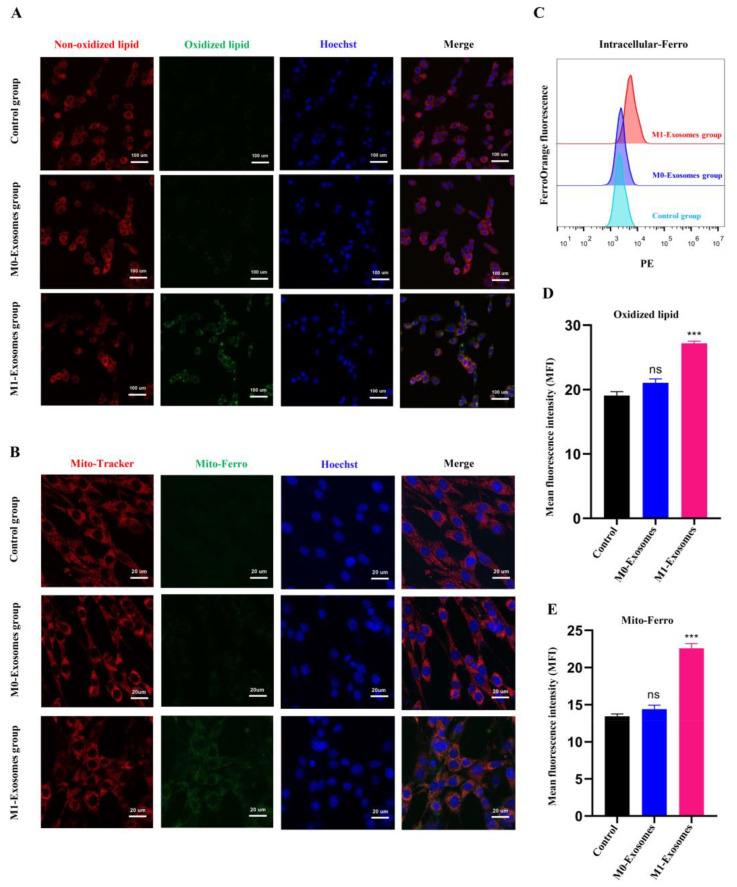
Levels of lipid peroxidation and mitochondrial and intracellular ferrous iron in neuronal cells following different treatments. (**A**) Representative fluorescent images of lipid peroxidation of neuronal cells after staining with C11BODIPY 581/591. Green represents oxidized lipid, red represents nonoxidized lipid, and blue represents Hoechst-stained nuclei. Scale bars = 100 μm. (**B**) Representative fluorescent images of mitochondrial ferrous iron obtained using Mito-Tracker (Red), Mito-Ferro (Green), and Hoechst (blue). Scale bars = 20 μm. (**C**) The intracellular ferrous iron level was determined using FerroOrange (PE fluorescence) with flow cytometry analysis. (**D**,**E**) Statistical results of lipid peroxidation and mitochondrial ferrous iron levels in neuronal cells (ns *p* > 0.05, *** *p* < 0.001). Data are presented as the mean ± SD of three independent experiments.

The fluorescent probe C11BODIPY581/591, Mito-FerroGreen, and FerroOrange were used to detect the levels of lipid peroxidation and mitochondrial and intracellular ferrous iron, respectively, in neuronal cells following different treatments. M1-BV2 exosomes increased the levels of lipid peroxidation (Figure 6A), mitochondrial ferrous iron (Figure 6B), and intracellular ferrous iron (Figure 6C) in neuronal cells, whereas M0-BV2 exosomes had no significant impact on the levels of ferrous iron and lipid peroxidation compared to controls (Figure 6A–E).

## 4. Discussion

Microglia show various phenotypes when responding to diverse environmental stimuli. Resting microglia are regarded as the M0 phenotype, while polarized microglia are classified into two subtypes: the M1 phenotype and the M2 phenotype [35,36]. It is notable that M1-polarized microglia accelerate chronic inflammation, oxidative stress, and accumulation of pathological proteins in chronic neurodegenerative diseases [37,38]. These microglia can secrete proinflammatory factors, leading to the destruction of neurons and ultimately the induction of neurodegeneration [39,40].

Exosomes play critical roles in cell-to-cell communication [41]. Although polarized microglia play a vital role in the development of neurological disorders [42,43], the effects of M1 microglia exosomes on ferroptosis in neurons have rarely been studied. As the standard primary microglia cell line, BV2 microglia are widely used in immunological discoveries [44,45], phagocytosis studies [46], and neurodegenerative disease research [47,48,49]. Here, we identified the gene expression profile of M0-BV2 and M1-BV2 microglial exosomes by high-throughput sequencing and revealed 1557 upregulated and 102 downregulated DEGs between the two exosome types. Importantly, the DEGs were shown to be significantly enriched in the ferroptosis pathway and inflammation-related pathways by enrichment analyses. In 2012, Doxin et al. [14] first identified a form of iron-dependent programmed cell death, termed ferroptosis, caused by lipoperoxidation damage of the membrane. There is compelling evidence that ferroptosis plays an important role in inflammation. For example, several antioxidants functioning as ferroptosis inhibitors have been shown to exert anti-inflammatory effects in experimental models of certain diseases [50]. It has also been established that ferroptosis cells trigger the innate immune system by releasing inflammation-linked damage-related molecules and that immune cells stimulate the inflammatory response by recognizing the operational mechanism of ferroptosis [51]. Recent studies have shown that ferroptosis plays a significant role in the pathogenesis of various neurodegenerative diseases [15,52]. Tuo et al. [53] demonstrated that ferroptosis can cause astroglia death after ischemic stroke, with ferroptosis inhibitors (ferroststatin-1 or liproxstatin-1) exerting protective roles in a murine ischemic stroke model.

Glutathione peroxidase GPX4 is a biomarker of ferroptosis that is usually inhibited during the ferroptosis process [33]. Hambright et al. [34] showed that GPX4 knockout in mouse brain neurons results in astroglia degeneration and cognitive impairment. In the present study, we cocultured M0-BV2 and M1-BV2 exosomes with neuronal cells. We found that the levels of the GPX4 were remarkably reduced in neuronal cells in the M1-Exosomes group compared to the M0-Exosomes group. SLC7A11 is one of the earliest identifiable regulators of ferroptosis, and is also responsible for maintaining redox homeostasis by participating in the metabolism of glutathione [14,54]. In the present study, there was no significant change in SLC7A11 expression in neuronal cells coincubated with M0-BV2 exosomes, whereas its expression was noticeably reduced in M1-BV2 exosome-treated neuronal cells. Polarized M1-BV2 microglia may reduce the expression of SLC7A11 and glutathione-dependent GPX4 via their secreted exosomes, thereby affecting glutathione metabolism. These findings suggest that inhibition of GPX4 and SLC7A11 expression could be one cause of ferroptosis in neuronal cells exposed to M1-BV2 microglial exosomes. Since ferroptosis is iron-dependent, and iron overload can induce ferroptosis, it is important to assess iron storage. FTH1 plays an important role in maintaining cellular iron balance during ferroptosis and is a promising target for neuroprotection [55,56]. FTH1, as a subunit of the iron storage protein ferritin, can effectively reduce the toxicity of iron via its ferroxidase activity [57,58]. To determine the effects of exosomes on iron metabolism, the level of FTH1 was assayed in the present study. M0-BV2 exosome treatment did not induce significant changes in the FTH1 level, whereas M1-BV2 exosome treatment significantly downregulated the FTH1 level in neuronal cells. This finding suggests that M1-BV2 microglial exosomes disrupt intracellular iron storage by regulating the expression of ferroptosis proteins, thereby inducing ferroptosis. These findings can serve as a basis for future research exploring the regulators in M1 microglial exosomes that result in ferroptosis and suggest novel therapeutic targets against neurodegenerative diseases.

Since ferroptosis is associated with lipid peroxidation [14] as well as intracellular and mitochondrial ferrous iron levels [59,60], we assessed the levels of lipid peroxidation and intracellular and mitochondrial ferrous iron in neuronal cells in the control, M0-Exosome, and M1-Exosome groups. The exosomes released by M1-BV2 microglia significantly increased ferrous iron and oxidative lipid levels in neuronal cells compared with the M0-BV2 exosomes. These results further support the finding that M1-BV2 microglia can induce ferroptosis of neuronal cells via their secreted exosomes. However, there is a need for future verification of the mechanisms identified in this study through primary microglia-derived exosomes and in vivo experiments.

In summary, our findings demonstrate that M1-BV2 microglial exosomes disrupt intracellular homeostasis of iron and lipid oxidation to induce ferroptosis. These findings offer new insights into the regulatory role of microglia-secreted exosomes in neuronal cells.

## 5. Conclusions

Exosomes derived from M1-polarized BV2 microglia can induce ferroptosis of neuronal cells, thereby aggravating neuronal damage. The findings of this study further the knowledge of the pathogenesis of neurological disorders. They also suggest that blocking the exosome release of M1-polarized microglia or targeting neuronal ferroptosis with ferroptosis inhibitors may offer new therapeutic avenues for treating neurodegenerative diseases.

## Figures and Tables

**Table 1:** Primer sequences for qRT-PCR.

Gene	Forward, 5′ → 3′	Reverse, 5′ → 3′
IL-1β	TGACGGACCCCAAAAGATGA	CTTGTTGATGTGCTGCTGCG
IL-6	GGATACCACTCCCAACAGACC	TGTTTTCTGCAAGTGCATCATCG
TNF-α	CGTCAGCCGATTTGCTATCT	CCTCAGGGAAGAATCTGGAAAG
GAPDH	AGGTCGGTGTGAACGGATTTG	TGTAGACCATGTAGTTGAGGTCA

## Data Availability

Not applicable.

## Supplementary Materials

The following supporting information can be downloaded at: https://www.mdpi.com/article/10.3390/cells11243956/s1, Table S1: Sequencing analysis data of M0-BV2 and M1-BV2 exosomes; Table S2: Differential upregulated genes; Table S3: Differential downregulated genes.
